# Effect of downscaling on the linearity range of a calibration curve in spectrofluorimetry

**DOI:** 10.1007/s00216-014-7844-2

**Published:** 2014-05-11

**Authors:** Radoslaw Kwapiszewski, Justyna Szczudlowska, Karina Kwapiszewska, Artur Dybko, Zbigniew Brzozka

**Affiliations:** Department of Microbioanalytics, Faculty of Chemistry, Warsaw University of Technology, Noakowskiego 3, 00-664 Warsaw, Poland

**Keywords:** Downscaling, Surface area to volume ratio, Fluorescence, Front-face illumination, Optical detection, Microfluidics

## Abstract

Interest in the microfluidic environment, owing to its unique physical properties, is increasing in much innovative chemical, biological, or medicinal research. The possibility of exploiting and using new phenomena makes the microscale a powerful tool to improve currently used macroscopic methods and approaches. Previously, we reported that an increase in the surface area to volume ratio of a measuring cell could provide a wider linear range for fluorescein (Kwapiszewski et al., *Anal. Bioanal. Chem.* 403:151–155, 2012). Here, we present a broader study in this field to confirm the assumptions we presented before. We studied fluorophores with a large and a small Stokes shift using a standard cuvette and fabricated microfluidic detection cells having different surface area to volume ratios. We analyzed the effect of different configurations of the detection cell on the measured fluorescence signal. We also took into consideration the effect of concentration on the emission spectrum, and the effect of the surface area to volume ratio on the limit of linearity of the response of the selected fluorophores. We observed that downscaling, leading to an increase in the probability of collisions between molecules and cell walls with no energy transfer, results in an increase in the limit of linearity of the calibration curve of fluorophores. The results obtained suggest that microfluidic systems can be an alternative to the currently used approaches for widening the linearity of a calibration curve. Therefore, microsystems can be useful for studies of optically dense samples and samples that should not be diluted.

**Figure:**
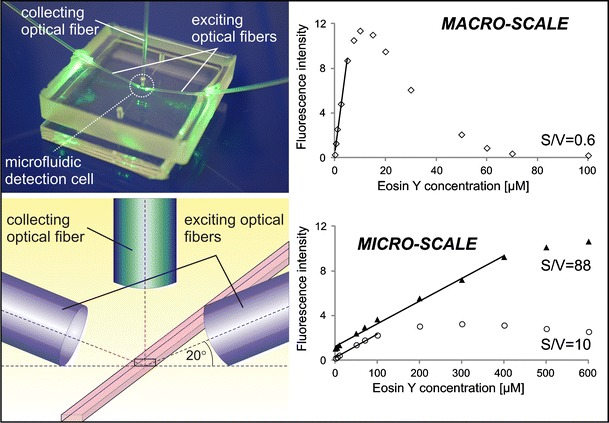
Microfluidic systems as a tool to increase the dynamic range of fluorophores

Microfluidic systems as a tool to increase the dynamic range of fluorophores

## Introduction

In recent years, microfluidic technology has provided novel and commercially successful products dedicated to the life sciences sector [1–3]. This is not surprising as miniaturization has a number of advantages, including working with smaller reagent volumes, better control of a process, shorter analysis time, and enhancing the high-throughput ability. However, the greatest potential of the microscale is in its physics [4]. Laminar flow, diffusion, fluidic resistance, the effect of the surface area to volume ratio (*S*/*V*), and surface tension are dominant effects at the microscale, whereas the influence of inertial and body forces is greatly reduced [4]. Microscale phenomena can be used to improve macroscale methods, and to perform experiments that are not possible at the macroscale. For example, microfluidic capillary electrophoresis devices, owing to their high *S*/*V*, allow minimization of Joule heating. In consequence, they allow the separation of compounds that cannot be separated by traditional capillary electrophoresis devices or high-performance liquid chromatography [5]. Another example is microchambers for cell culture, which mimic in vivo conditions much better than standard cell culture plates. The high *S*/*V* of such microchambers provides more effective oxygen supply and prevents dilution of secreted molecules. Cells growing within the microchambers can better control their environment by secretion and autocrine and paracrine signaling [6].

We previously studied the effect of a high *S*/*V* on fluorescence intensity [7]. We found that modification of a microchannel’s geometry to increase *S*/*V* provided a wider linear concentration range for fluorescein. In the work discussed in this article, we studied the effect of *S*/*V* on the fluorescence intensity of various fluorophores with a small and a large Stokes shift. We examined the effect of the geometry of the microfluidic detection cell (the effect of sample geometry), and the effect of concentration on the emission spectrum. We confirm the effect of *S*/*V* on the linearity range of the calibration curve of the fluorophores analyzed.

## Materials and methods

### Chemicals

4-Methylumbelliferone (4-MU) and quinine sulfate dihydrate were purchased from Fluka (Poznan, Poland). Fluorescein sodium salt, eosin Y, glycine, phosphate-buffered saline (PBS), citric acid, sodium phosphate dibasic, and sodium hydroxide were purchased from Sigma-Aldrich (Poznan, Poland). Sulfuric acid was purchased from POCH (Gliwice, Poland).

Investigations were conducted with the following fluorophore solutions: (1) 4-MU in 0.05 M citrate–phosphate buffer, pH 4.5, *λ*_ex_ = 320 nm, *λ*_em_ = 445 nm (protonated form of 4-MU); (2) 4-MU in 0.1 M glycine–NaOH buffer, pH 10.2, *λ*_ex_ = 365 nm, *λ*_em_ = 445 nm (deprotonated form of 4-MU); (3) quinine in 0.05 M sulfuric acid, *λ*_ex_ = 250 nm, *λ*_em_ = 445 nm; (4) fluorescein in 0.01 M PBS, *λ*_ex_ = 494 nm, *λ*_em_ = 521 nm; and (5) eosin Y in 0.01 M PBS, *λ*_ex_ = 521 nm, *λ*_em_ = 545 nm.

### Geometry and fabrication of the microfluidic detection cells

**Fig. 1:**
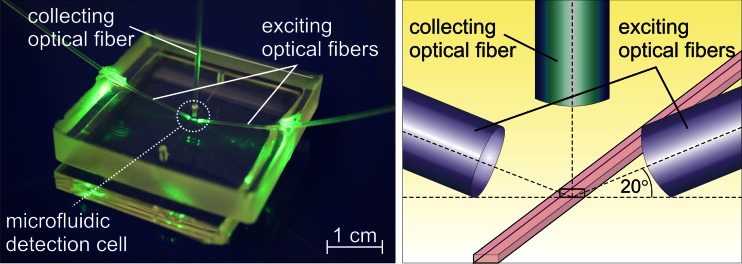
Photograph (*left*) and schematic view (*right*) of the microfluidic detection cell

The microfluidic detection cells were straight microchannels of rectangular cross section fabricated in poly(dimethylsiloxane) by the replica molding technique using micromilled poly(methyl methacrylate) masters. Each microfluidic cell was connected to a spectrofluorimeter (FluoroMax-3, Jobin Yvon, France) via optical fibers (600/950 μm, numerical aperture 0.22) embedded in poly(dimethylsiloxane). Two exciting fibers were placed at an angle of 20° to the bottom of the microchannel, whereas the collecting fiber was placed orthogonally to the microchannel. A photograph and a schematic view of the fabricated microfluidic detection cell are presented in Fig. 1. More details on the positioning of the optical fibers and illumination of the detection cells were presented in our previous report [7].

In this work, we used microfluidic detection cells of different geometries while keeping the same *S*/*V* of 10 mm^-1^, and cells with different values of *S*/*V* ranging from 10 to 88 mm^-1^ (see Table 1).

**Table 1:** Dimensions and surface area to volume ratios (*S*/*V*) of the microfluidic detection cells

Microfluidic detection cell	Microchannel width (μm)	Microchannel height (μm)	*S*/*V* (mm^-1^)
1	400	400	10
2	1,000	250	10
3	250	1,000	10
4	250	250	16
5	250	100	28
6	250	50	48
7	250	25	88

### Macroscale measurements

Measurements of the fluorescence intensity of the selected fluorophores at the macroscale were performed in a standard cuvette (*S*/*V* = 0.6 mm^-1^) that was illuminated centrally and observed at a right angle by using a spectrofluorimeter (FluoroMax-3, Jobin Yvon, France).

First, the effect of concentration on the emission spectra of a protonated and a deprotonated form of 4-MU, quinine, fluorescein, and eosin Y at concentrations of 0.1, 1.0, 10, and 100 μM was examined.

Next, the calibration curves for each fluorophore ranging from 0.01 to 1,000 μM were constructed, and the limits of linearity were found (using MicroCal Origin). The acceptance criterion for the correlation coefficient, *r*, as 0.995 (*r* ^2^ = 0.99) was set to determine the limits of linearity.

### Microscale measurements

Three microfluidic detection cells having the same *S*/*V* but different geometries (see Table 1) were used to study the effect of the sample geometry on the emission intensity of fluorescein as a model fluorophore. Measurements were performed for concentrations of fluorescein ranging from 0.01 to 500 μM.

Two microfluidic detection cells having *S*/*V* of 10 and 88 mm^-1^ (see Table 1) were used for investigations on the effect of concentration on the emission spectra of fluorophores with a large and a small Stokes shift. Measurements were performed at concentrations of 0.1, 1.0, 10, and 100 μM for each fluorophore.

Finally, five microfluidic detection cells with different *S*/*V* values (see Table 1) were used to construct the calibration curves of the fluorophores ranging from 0.01 to 1,000 μM. The limits of linearity of the constructed calibration curves were found using the same acceptance criterion as for the macroscale measurements (*r* ^2^ = 0.99) using MicroCal Origin.

The solutions of fluorophores were introduced into the microfluidic cells at a flow rate of 10 μL min^-1^ using a syringe pump (NE 1000 New Era Pump Systems, Farmingdale, NY, USA). The flow analysis allowed us to eliminate the possible effect of photobleaching, particularly observable in the case of the fluorophores with a small Stokes shift.

## Results and discussion

### Effect of sample geometry

Different configurations of the detection cell (variations in width and height) were studied. The limits of linearity for fluorescein determined in the 400/400 (a microchannel 400 μm wide and 400 μm high), 250/1,000, and 1,000/250 microfluidic detection cells (all having the same *S*/*V* of 10 mm^-1^) were 200, 200, and 150 μM, respectively. The values obtained are quite similar. This suggests a lack of a significant effect of the sample geometry on the measured signal. However, the fluorescence signal determined is not solely the result of the dimensions of the microfluidic channel. The signal also depends on the type, the size, and the arrangement of the optical fibers used. In this work, optical fibers with a core diameter of 600 μm were used. In the case of the 400- and 250-μm-wide microchannels, the core allowed us to collect the signal from their entire widths, whereas in the case of the 1,000-μm-wide microchannel, the signal was collected only from the central region. For all the microchannels, a nonuniform distribution of fluorescence caused by a primary inner-filter effect was observed. The excitation radiation was mostly absorbed by the concentrated solutions of the fluorophore at the surface facing the light [8, 9]. In our opinion, the lower value of the limit of linearity observed for the widest microchannel could be the result of our collecting the fluorescence at the point where the intensity of excitation was diminished.

From these results, it can be concluded that the change in the geometry of the microchannel while keeping the same *S*/*V* does not significantly affect the measured fluorescence signal. However, it is important to have the same measurement conditions for each detection cell, including the proper kind, size, and arrangement of optical fibers. It is also important to be aware of the possibility of the occurrence of inner-filter effects for certain configurations of microfluidic cells.

### Effect of concentration on the emission spectra of the fluorophores

The wavelengths of the emission maximum in the fluorescence spectra of quinine, 4-MU at pH 4.5, and 4-MU at pH 10.2, irrespective of concentration and *S*/*V* of the cell, were not shifted (data not shown). The results confirm that fluorophores with a large Stokes shift are not sensitive to the phenomenon of reabsorption of the emitted light [9], and, therefore, the increase in concentration of such fluorophores does not result in shifting of the fluorescence wavelength to higher wavelengths.

However, for fluorophores whose absorption and emission spectra overlap, a dramatic effect of concentration on the emission spectrum can be seen [9]. As presented in Table 2, the shift in the emission maxima of fluorescein and eosin Y is significant, particularly in the case of the measurements in a cuvette. For fluorescein, the emission maximum shifts from 511 nm for a concentration of 0.1 μM to 536 nm for 100 μM (in a cuvette). In the case of eosin Y, the shift is from 536 to 586 nm. The shift in the emission maximum is smaller, the greater the value of *S*/*V* of the microfluidic detection cell. The emission maximum for fluorescein measured in the microfluidic cell having *S*/*V* of 10 mm^-1^ shifts from 511 to 525 nm, whereas the shift in the microfluidic cell having *S*/*V* of 88 mm^-1^ is very small (from 510 to 512 nm). A similar trend was observed for eosin Y. In the case of the measurements with the microfluidic cells having *S*/*V* of 10 and 88 mm^-1^, the emission maximum shifted from 534 to 556 nm and from 534 to 536 nm, respectively.

**Table 2:** Wavelengths (nm) of the maximum fluorescence for the fluorophores with a small Stokes shift at different concentrations

Compound	*S*/*V* (mm^-1^)	Concentration (μM)			
		0.1	1.0	10	100
Fluorescein	0.6 (cuvette)	511	514	522	536
	10	511	513	520	525
	16	510	512	513	518
	28	510	511	512	515
	48	510	510	511	513
	88	510	510	511	512
Eosin Y	0.6 (cuvette)	536	540	549	586
	10	534	534	542	556
	16	534	534	537	542
	28	534	534	537	539
	48	534	534	535	538
	88	534	534	534	536

A possible explanation for this phenomenon is the similarity of the measurement setup (a microchannel with a high *S*/*V*, and the special arrangement of the optical fibers) for the front-face illumination geometry using either triangular cuvettes or square cuvettes oriented at 30° to 60° relative to the incident beam [9–11]. Fluorescence observation from the side of excitation, i.e., from the front face, is the commonest method to minimize the possible effect of reabsorption [10]. In such an arrangement, the excitation beam is not so attenuated by the solution, and thus more light reaches the region that is viewed by the optics of the detection system. In the case of the cell having *S*/*V* of 88 mm^-1^, complete absorption of the excitation light at the surface could occur, and almost no shift of the wavelength of the emission maximum in the fluorescence spectra of fluorescein and eosin Y was observed. In the case of the other microfluidic detection cells, penetration of the excitation light into the bulk of the solution could occur, and thus we observed a shift of the emission spectra to higher wavelengths. The shift was larger, the greater the height of the microchannel.

### Effect of S/V on the linear range of the fluorophores

The limits of linearity of the fluorophores with a large and a small Stokes shift measured in a standard cuvette and in the microfluidic detection cells are presented in Table 3.

**Table 3:** The limits of linearity of the fluorophores measured in a cuvette and in the microfluidic detection cells with different *S*/*V* values

Compound	*S*/*V* (mm^-1^)	Limit of linearity (μM)
Fluorescein	0.6 (cuvette)	10
	10	200
	16	200
	28	250
	48	350
	88	600
Eosin Y	0.6 (cuvette)	5
	10	100
	16	100
	28	125
	48	175
	88	400
Quinine	0.6 (cuvette)	10
	10	400
	16	400
	28	450
	48	500
	88	600
4-MU, pH 4.5	0.6 (cuvette)	15
	10	600
	16	625
	28	650
	48	700
	88	800
4-MU, pH 10.2	0.6 (cuvette)	10
	10	400
	16	450
	28	500
	48	700
	88	1,000

*4-MU* 4-methylumbelliferone

**Fig. 2:**
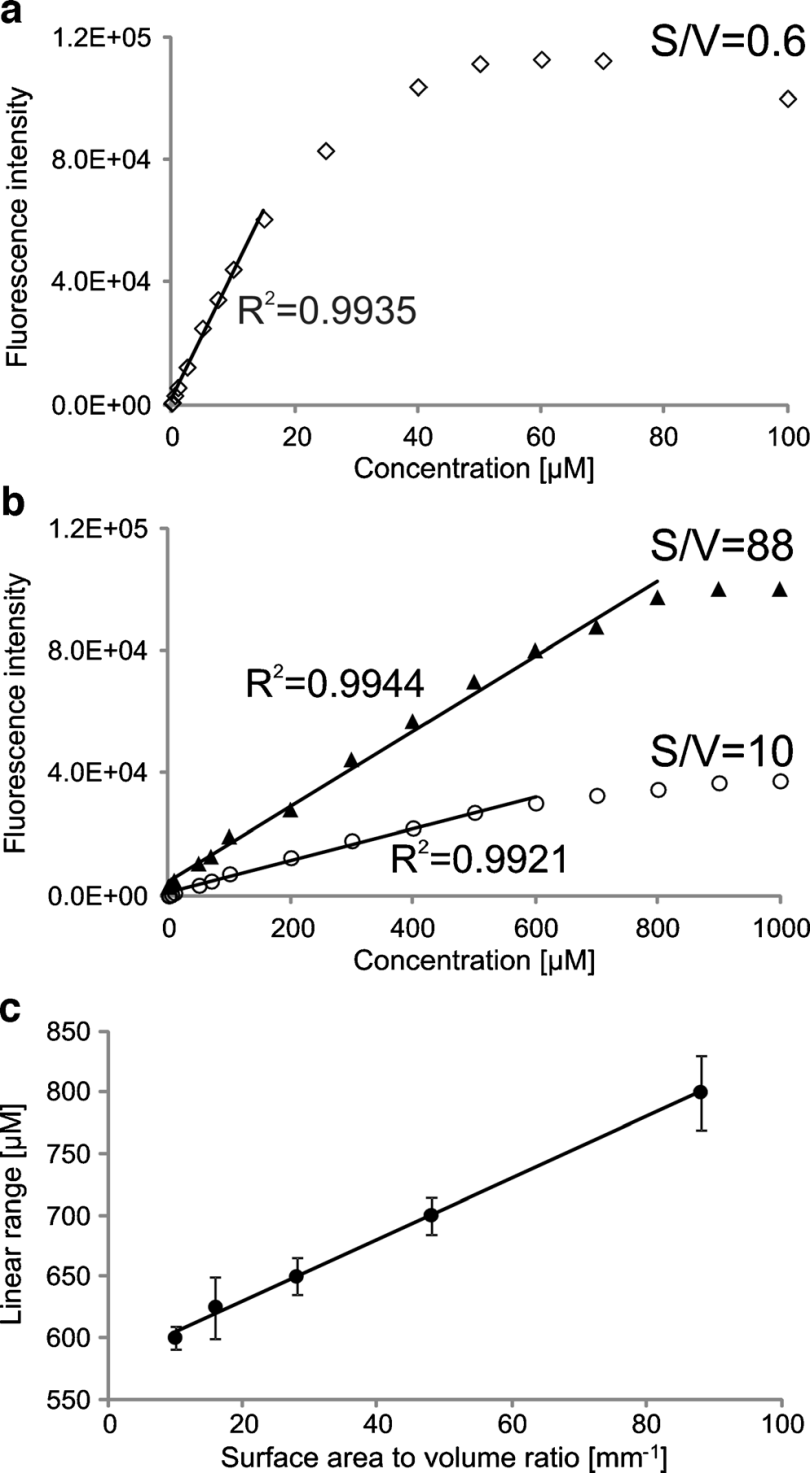
Effect of concentration on the fluorescence intensity of 4-methylumbelliferone at pH 4.5: (**a**) macroscale experiment (measurement in a 1 cm × 1 cm cuvette; surface area to volume ratio, *S*/*V*, of 0.6 mm^-1^), and (**b**) microscale experiment (measurements using microfluidic cells with *S*/*V* of 10 and 88 mm^-1^). (**c**) Linear dependence of the limit of linearity on *S*/*V*

**Fig. 3:**
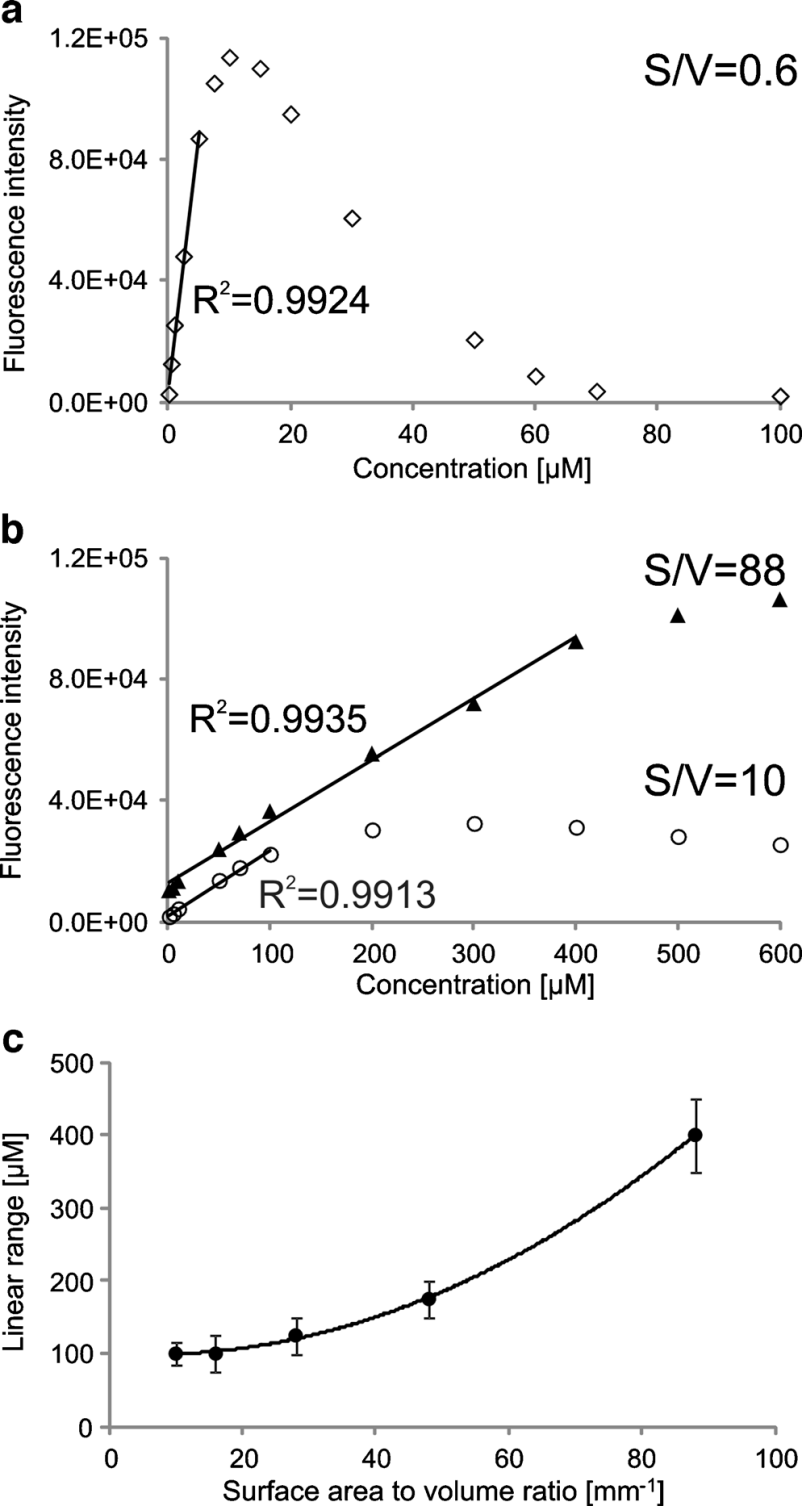
Effect of concentration on the fluorescence intensity of eosin Y: (**a**) macroscale experiment (measurement in a 1 cm × 1 cm cuvette; *S*/*V* = 0.6 mm^-1^), and (**b**) microscale experiment (measurements using microfluidic cells with *S*/*V* of 10 and 88 mm^-1^). (**c**) Exponential dependence of the limit of linearity on *S*/*V*

Increasing *S*/*V* of the detection cell resulted in a higher limit of linearity for each fluorophore. Comparing the results between the cuvette-based measurements and the measurements at the microscale, we can seen that the limit of linearity results differ by at least one order of magnitude. The total optical density of the fluorophores at the microscale could be many times larger [9]. Figures 2 and 3 present results obtained for 4-MU at pH 4.5 and eosin Y, respectively. In the case of 4-MU at pH 4.5, the limit of linearity of the calibration curve in a standard cuvette was at least 40 times lower than in the microfluidic cells. A linear dependence of the limit of linearity on *S*/*V* of the microfluidic detection cell was found. The linear growth model was assumed and was fitted to the data points for all fluorophores with a large Stokes shift analyzed in this work (using MicroCal Origin).

In the case of eosin Y, the limit of linearity of the calibration curve in a standard cuvette was at least 20 times lower than in the microfluidic cells. For eosin Y and fluorescein, an exponential dependence of the limit of linearity on *S*/*V* of the microfluidic detection cell was found (using MicroCal Origin).

The difference between these two models (i.e., the linear model to fit the data points for the fluorophores with a large Stokes shift and the exponential growth model for the fluorophores with a small Stokes shift) obtained is probably attributed to the reabsorption effect described earlier. Because the excitation and emission wavelengths for each fluorophore were set (see “Chemicals”) and not changed during the measurements in a cuvette and in the microfluidic detection cells, the registered fluorescence signal was lower when the emission spectra shifted to higher wavelengths. For example, the emission wavelength of eosin Y, according to the literature [12], was set to 545 nm. The wavelength of the maximum fluorescence for eosin Y at a concentration of 100 μM in a cuvette was 586 nm, whereas in the microfluidic cell having *S*/*V* of 88 mm^-1^ it was 536 nm. Thus, the detected signal at the microscale was higher, as the maximum-fluorescence wavelengths in the microfluidic cells were closer to the initially set value of the emission wavelength of 545 nm. These results suggest that the observed higher limits of linearity at the microscale are only the result of shifting of fluorescence spectra. However, the assumption that the concentration-dependent shift of fluorescence spectra was the only determinant for the extension of the limits of linearity was not confirmed by the results obtained for the fluorophores with a large Stokes shift, for which the reabsorption effect does not occur. In the case of the two forms of 4-MU and quinine, the initially set value of the emission wavelength corresponds to the maximum-fluorescence wavelength obtained in a cuvette and in the microfluidic detection cells. The results for the fluorophores with a large Stokes shift suggest that an additional effect or phenomenon related to *S*/*V* of the cell occurs. High fluorophore concentrations can result in quenching due to interactions, such as radiative and nonradiative transfer and excimer formation [9]. In our opinion, increasing *S*/*V* increases the probability of collisions between molecules and cell walls with no energy transfer. Hence, the possible effect of concentration self-quenching, which affects the limit of linearity and depends on collision between fluorophore molecules, in cells with high *S*/*V* can be reduced.

## Conclusions

Gaining knowledge of fluorescence detection at the microscale and exploration of new effects and phenomena in fluorimetric measurements is very important. Firstly, currently existing methods and approaches can be improved, and secondly, mistakes during development of new procedures and strategies can be avoided.

In the work discussed here, the effect of downscaling of the detection cell on the limit of linearity of the calibration curve of various fluorophores was studied. We analyzed fluorophores with a large Stokes shift, such as quinine and a protonated and a deprotonated form of 4-MU, and fluorophores with a small Stokes, such as fluorescein and eosin Y. We found that a change in the geometry of the measuring cell while maintaining a constant *S*/*V* does not affect the detected fluorescence signal. Moreover, we observed a significant effect of reabsorption for the fluorophores with a small Stokes shift, and the possibility of elimination of this effect when using microfluidic cells with high *S*/*V*. The reabsorption effect was almost eliminated when a microfluidic cell having *S*/*V* of 88 mm^-1^ was used. We also observed that the limits of linearity of the fluorophores using a standard cuvette were more than one order of magnitude lower than in the microfluidic cells. The geometry of the optical detection cells presented here has much in common with the front-face illumination geometry which is typically used to obtain a wider linear range of a calibration curve. The special geometry of a microfluidic channel and a proper arrangement of optical fibers can result in minimization of inner-filter effects.

Microfluidic systems with optical detection can be an alternative to currently used different geometric arrangements of cuvettes or to using mathematical corrections [9] to minimize the effect of reabsorption. Such microsystems can be useful for studies of optically dense samples such as whole blood, or highly scattering solutions [13]. The approaches to increase the dynamic range of fluorophores are also important when dilution of an analyzed sample may cause changes in solvation, conformation, binding, degree of association, and other features [8]. Finally, the effect of high *S*/*V* of a detection cell on the fluorescence signal should be taken into consideration when performing measurements based on fluorescence quenching, i.e., study of conformational and/or dynamic changes of proteins in complex macromolecular systems, or study of the interactions of quenchers with proteins, membranes, and nucleic acids [9]. The effect described should also be taken into account when developing miniaturized tools using fluorescence detection. Often, to verify the protocol or method developed, microscale results are compared with macroscopic results. It is important to take into consideration the effects characteristic for the microscale. They may cause significant divergences between the results obtained and lead to wrong conclusions.
